# Integrating augmented reality in language learning: pre-service teachers’ digital competence and attitudes through the TPACK framework

**DOI:** 10.1007/s10639-022-11123-3

**Published:** 2022-05-27

**Authors:** Jose Belda-Medina, José Ramón Calvo-Ferrer

**Affiliations:** Department of English Studies, Digital Language Learning (DL2), University of Alicante, Alicante, Spain

**Keywords:** Augmented reality (AR), Language learning, Pre-service teachers, Digital competence, Attitudes

## Abstract

Although the use of Augmented Reality (AR) in language learning has increased over the last two decades, there is still little research on the preparation of pre-service teachers as AR content creators. This paper focuses on analyzing the digital competence and attitudes of teacher candidates to integrate AR in the foreign language classroom. For this purpose, eighty-five college students were assigned into different teams to create their own AR-based projects which aimed at teaching English and content to young learners. The teacher candidates employed several software development kits (SDKs) to construct collaborative AR projects in a five-week period, including discursive and illustrative representations of the learning content. In this research based on a mixed method, quantitative and qualitative data were gathered trough AR project presentations and surveys encompassing two validated scales, the Technological Pedagogical Content Knowledge (TPACK) framework and the Augmented Reality Applications Attitudes Scale (ARAAS). The statistical data and qualitative findings revealed that the participants lacked practical knowledge on AR content creation and implementation in Education. The major problems were related to the TPK (Technological Pedagogical Knowledge) intersection since participants had been previously trained in AR technology just as recipients and not as content creators and educators.

**Supplementary Information:**

The online version contains supplementary material available at 10.1007/s10639-022-11123-3.

## Introduction

The use of Augmented Reality (AR) has increased over the last two decades thanks to the widespread of portable electronic devices, such as tablets and smartphones, and the emergence and availability of free or low-cost AR tools. The adoption of AR has been particularly prolific in digital gaming and education, which has resulted in a rich body of literature in both areas as illustrated in systematic reviews (Li et al., 2017; Cabero-Almenara et al., 2019; Parekh et al., 2020; Sırakaya & Alsancak Sırakaya, 2020; Li & Wong, 2021). However, the implementation of this breakthrough technology is still in its infancy, as explained by different authors who investigated the benefits and obstacles of integrating AR in Education (Saidin et al., 2015; Yuliono, 2018).

AR can be defined as an interactive technology that combines the physical world with computer-generated elements in real time providing an enhanced immersive experience with superimposed digital content (3D images, sound, text). Originally, Azuma (1997) described it as a system including three key elements: a combination of real and virtual content, the interaction in real time and the registration in 3D. This technology is rapidly evolving as regards AR visualization and development tools and as a consequence, there is nowadays a good number of AR apps and wearables in the market, partly due to the pervasiveness of smartphones. In fact, mobile AR is one of the fastest-growing sectors with an impact on Education (Giannakas et al., 2018; Hedberg et al., 2018).

The three types of AR technology commonly referred to are marker-based, markerless and location-based, although some authors reduce them to two: vision-based (marker-based and markerless) and location-based. All of them have been integrated with different levels of success in several educational contexts (Georgiou & Kyza, 2018; Lee, 2020). There is also a wide range of software development kits (SDKs) available on the web with different features (3D object tracking, Cloud-storage, Geolocation, etc.), which can be used for artifact creation such as Augmania, Blippar, Vuforia, Wikitude or Zapworks. The availability of these SDKs is an advantage since users may become content creators rather than just AR recipients.

However, the integration of this new technology poses several challenges concerning AR knowledge and training to current and future educators. There are several quantitative and qualitative studies about in-service teachers’ readiness to adopt AR in the general education setting (Jwaifell, 2019; Sáez-López et al., 2020; Wei et al., 2021) and on students’ attitudes toward AR in different disciplines (Cheng, 2017a; Sirakaya & Kiliç Çakmak, 2018). Nevertheless, no study to date has been found specifically about the technological and pedagogical preparation of future educators to integrate AR in the Content and Language Integrated Learning (CLIL) classroom. The novelty of this research lies in analyzing the impact of developing AR collaborative projects on pre-service teachers’ digital and pedagogical skills in the CLIL classroom. The analysis is based on the correlation of two validated scales, the TPACK framework and the Augmented Reality Application Attitudes (ARAAS) scale. This is relevant as teacher candidates will be responsible for AR adoption in Education over the next years.

## Literature review

### AR in content and language integrated learning (CLIL)

Several works have come out to light about the use of AR in language learning from different perspectives. Khoshnevisan and Le (2018) reviewed 19 empirical studies dedicated to AR integration in language learning and highlighted some benefits (enhanced motivation, decreased cognitive load, improved language performance and creativity) and constraints (technical issues with AR apps, availability of AR materials, cost-effectiveness, teachers’ attitudes and lack of training) in Education.

Similarly, Fan et al. (2020) analyzed 53 papers from 2010 to 2019 about AR in early language learning and identified three main learning activities (word spelling games, word knowledge activities and location-based word activities) and five design strategies (3D multimedia content, hands-on interaction with physical learning materials, gamification, spatial mappings, and location based features). The authors also suggested two areas for further research: ‘(a) understanding the learning effect of various design strategies and (b) investigating the generalization and maintenance of AR learning gains as well as the effectiveness of instruction with AR applications’ (p. 1092).

More recently, Norzaimalina et al. (2021) reviewed 29 works from 2010 to 2020 on AR usage in language learning and noticed a sharp increase in the number of publications over the last four years, particularly in the field of mobile AR and gamification. These authors emphasized the need for further research on cognitive processes, knowledge building and teacher and student collaboration. Similarly, Huang et al. (2021) examined 88 studies on AR and VR in language learning indicating the main benefits of using both state-of-the-art technologies in the classroom such as immersive experience, reduced language anxiety, enhanced motivation, improvement of learning outcomes and positive perception. These authors also stressed the need to provide proper training for teachers and to explore variables such as student engagement and satisfaction.

One potential field of interest about AR implementation is Content and Language Integrated Learning (CLIL), an umbrella term used to describe modern practices in bilingual education aimed at learning associatively a content subject and a foreign language. Although the CLIL foundations can be traced back to previous models of language immersion programs in Canada and the USA, this approach has become popular in some European countries over the last two decades. After its inception in the early 1990s (Marsh, 1994), this method was further developed through several practices and principles such as instructional scaffolding or ‘the 4 Cs’ (Content, Communication, Cognition and Culture), which have been examined in a variety of works (Coyle, 2007; Mahan, 2020).

In fact, AR technology can be very useful in CLIL since it combines language and content learning. However, studies on AR adoption in this area remain very scarce to date. Expósito et al. (2017) reported the affordances of using AR in a Natural Science class with Elementary Education students such as the learning of abstract concepts through 3D images, enhanced communicative competence and decreased language anxiety. Merzlykin et al. (2018) investigated the integration of this technology in Secondary Education and pinpointed that AR helps to get some practical experience in using a foreign language and understand abstract concepts.

### AR in teacher training programs and the TPACK framework

Different studies have underlined the need for better training in AR technology among current and future educators. Some authors have even identified certain predictors for teacher readiness and willingness to use AR such as prior knowledge (Chang et al., 2020), motivation (Cheng, 2017b; Georgiou & Kyza, 2018) and positive attitudes (Wojciechowski & Cellary, 2013). However, AR teacher training is still an area to explore since most works used research participants as mere recipients. Thanks to the wide range of SDKs available today, teachers and learners alike can design their own projects and integrate them in the classroom but this requires some technical and pedagogical preparation.

One of the major difficulties in integrating AR is the lack of knowledge on theories and pedagogical principles among pre- and in- service teachers (Kerr & Lawson, 2020). The three most commonly cited approaches for AR integration are Constructivism, Connectivism and Situated Learning (Dunleavy & Dede, 2014; Zhang et al., 2020). First, the Constructivist approach upholds that learning is a social activity where learners actively construct their knowledge through previous experience and interaction with new events. According to Zhang et al. (2020: 2019), ‘learners can acquire contextualized linguistic and content knowledge from the AR-based language learning materials, internalize and construct the knowledge, and then use the obtained knowledge in productive tasks’. Then and closely related with Constructivism, the Situated Learning Theory (SLT) claims that learning takes place within a community of practice and that knowledge should be acquired in the environment in which it is applied, according to three of its main principles: context, interaction and communication. In this regard, AR technology provides different possibilities for context-aware learning in lifelike situations since it turns the surrounding environment into a digital experience by placing virtual objects in the real world (Wen & Looi, 2019). Finally, Connectivism is a relatively new theory (Siemens, 2004) that supports digital learning and social collaboration among students, who become part of a network and learn when they make connections and links. According to this theory, social media, gamification and simulation are key factors to consider in the classroom. For example, Techakosit and Wannapiroon (2015) evaluated the Connectivism learning environment in an AR science laboratory to enhance scientific literacy.

In regard to teacher training, some studies have delved into pre-service teachers’ readiness and attitudes toward AR integration in different disciplines using various models. Kim and Curry (2020) employed the ADDIE (Analyze, Design, Develop, Implement and Evaluate) model to evaluate undergraduate students’ preparation to create and integrate AR projects in the classroom. In the EFL/ESL field, Okumuş (2021) analyzed the AR perception, acceptance and self-efficacy level among 50 language teacher candidates using the Technology Acceptance Model (TAM). For this purpose, the participants had to design AR enhanced activities to teach English. The results revealed a high acceptance level of AR for designing language learning materials.

The TPACK framework, as originally envisaged by Shulman (1986) and later complemented by Mishra and Koehler (2006), can be used as an effective tool to measure EFL pre-service teachers’ readiness to integrate AR in the CLIL classroom. This framework is based on a construct of three different dimensions: Content Knowledge (CK), Pedagogical Knowledge (PK) and Technological Knowledge (TK), and on their intersections (TCK, TPC, PCK, TPACK). In this model, CK refers to ‘what’ we teach (subject content), PK is related to ‘how’ we teach it (methods) and TK is associated with the tools (technology) we use to teach it (content), thus indicating a correlation between the three dimensions. TPACK has been extensively used as a measurement tool in several disciplines at different educational levels. For example, Jwaifell (2019) examined the aptitude of 60 in-service Science teachers to integrate AR through the TPACK framework and revealed that female teachers were more ready than males to adopt this technology and that the years of teaching experience did not affect their AR readiness. Jang et al. (2021) employed TPACK and an extended Technology Acceptance Model (eTAM) in their study on 292 in-service teachers’ willingness to integrate AR and VR technologies. They found significant correlations between teachers’ attitudes and intentions, and concluded that ‘positive attitudes toward AR and VR-enabled instruction have an effect on their continuous use in the classroom’ (p. 6806).

However, the studies published on AR to date using TPACK have mainly focused on in-service teachers and they have been applied in other disciplines, so there is a need to analyze the technological and pedagogical preparation as well as the attitudes toward AR among language teacher candidates in the CLIL classroom through this holistic framework.

## Research objectives and questions

The objectives of this paper are three:

1. To examine pre-service teachers’ knowledge on AR for language and content learning in the CLIL classroom.
2. To measure pre-service teachers’ technological and pedagogical skills regarding the integration of AR in a CLIL classroom through the TPACK framework
3. To analyze pre-service teacher’s attitudes toward the integration of AR in language learning (ARAAS scale) and any existing correlation with their technological and pedagogical skills (TPACK).

These objectives are related with the following research questions:

1. What knowledge do teacher candidates have about the use of AR in education?
2. What technological and pedagogical skills do teacher candidates have to integrate effectively AR in the CLIL classroom?
3. What are the teacher candidates’ attitudes toward the adoption of AR after developing their own collaborative AR projects?

## Method

### Participants and context

The research was based on a convenience sampling method. Initially, there were 92 undergraduate students enrolled in the Applied Linguistics class taught by the authors in a medium-sized university in Spain. The students were officially divided into two groups of approximately 45 participants that would meet twice a week during two-hour class sessions. Seven students dropped the class during the first two weeks and were excluded from the research. The remaining 85 students completed all the tasks and were randomly arranged in small teams (4–5) in order to create collaborative AR projects that aimed at teaching English to children in a CLIL classroom. This digital project was part of their evaluation. Their English language level corresponded to a B2 or higher according to the CEFR framework, and they all lacked any previous training in AR content creation or implementation. The last unit in the subject was devoted to CLIL as a dual-focused educational approach and it covered different aspects such as its origins and aims, principles and strategies, types and models, benefits and challenges, and modern practices. The ages of all participants were aged between 22 and 30, 82% were female and 18% were male students. Written informed consent was obtained from all participants, including the use of data analysis and images of their class projects for scientific purposes. The research procedure followed the instructions provided by the Ethical Review Board of the University of Alicante available on its website https://bit.ly/3PpTiRV.

## Research instruments and procedure

The research design was based on a mixed method in order to collect both quantitative and qualitative data (Riazi, 2017). The experiment took place over a five-week period and comprised ten two-hour sessions. Quantitative and qualitative data were obtained through a pre-post-test, class observation and group discussion. First, a group of 85 language teacher candidates were pre-tested regarding their previous experience and knowledge on AR technology and CLIL principles and methods. Then, they read different articles and completed several Moodle tasks about CLIL and received a theoretical session about AR technology in language learning. Secondly, the participants were arranged in teams of 4–5 members and agreed on the topic and setting for their AR project; they could choose any topic included in the curriculum but they needed to consider the target students, language level, setting, etc. Later on, all participants received a practical session on different concepts (trigger, overlay, tracking, 3D, scan, extended tracking, markerbased, markerless, geolocation) and SDKs in AR technology (Zapworks, Roar, Vuforia, Wikitude, Aumentaty, etc). Then, each team selected the AR tool that best fitted their needs after considering different factors (operating system, AR type, interface, pricing, publishing, limitations).

**Fig. 1:**
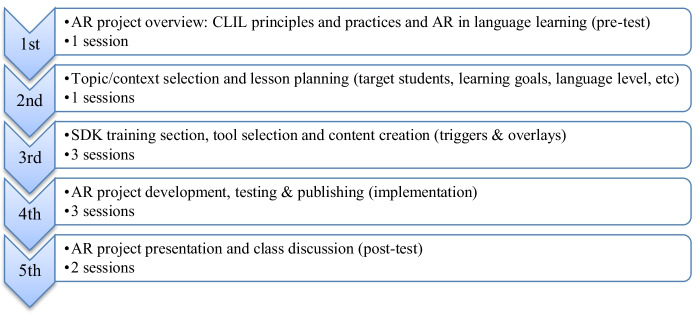
Research stages included in the AR project (10 two-hour sessions)

In a later stage, the teams developed their AR collaborative projects following the instructions previously provided: the projects needed to include a minimum of ten multimedia activities, which should be meaningful, organized in a sequential manner and specifically designed to learn content and language about the topic they had selected. In the final stage, the different teams shared their projects and explained the topic, target students, multimedia activities and learning goals to the rest of the class, and participated in a semi structured discussion about the benefits and limitations of AR in the CLIL classroom after their experience as content creators. Then, the teacher candidates were post-tested using the TPACK framework, which was partly adapted to our research objectives (Appendix). Besides, the participants’ attitudes toward AR were measured through the Augmented Reality Applications Attitude (ARAAS) scale, which was originally designed by Küçük et al. (2014) and later complemented by Díaz Noguera et al. (2017). Quantitative data was analyzed through the IBM SPSS Statistics 22 software. Figure 1 summarizes the different research stages.

In relation to the TPACK framework, CK in this experiment refers to the knowledge on CLIL concepts and principles. PK is related to the knowledge on the different pedagogical methods and practices in the CLIL classroom and TK is associated with the knowledge on general technology and AR, both for content creation (use of SDKs) and implementation (visualization). These main TPACK sections and intersections are summarized in Table 1.

**Table 1:** TPACK main sections and intersections related with AR technology in the CLIL classroom

	Dimension	Descriptor	Items
CK	Content Knowledge	Knowledge on ELF and CLIL principles and theories	5
PK	Pedagogical Knowledge	Knowledge on language teaching methods in the foreign language classroom	5
TK	Technological Knowledge	Knowledge on technology in general and AR	6
PCK	Pedagogical Content Knowledge	Knowledge on CLIL methods and practices to teach and evaluate EFL students	5
TPK	Technological Pedagogical Knowledge	Knowledge on how to use different pedagogical methods and approaches for AR integration in the CLIL classroom	6
TCK	Technological Content Knowledge	Knowledge on how to use AR technology, both as visualization and creation (types, tools, etc.)	3
TPACK	Technological Pedagogical and Content Knowledge	Knowledge on how to use pedagogical methods and integrate AR technology to teach content and language in the CLIL classroom	3

## Results and discussion

### AR projects and pre-test results

**Fig. 2:**
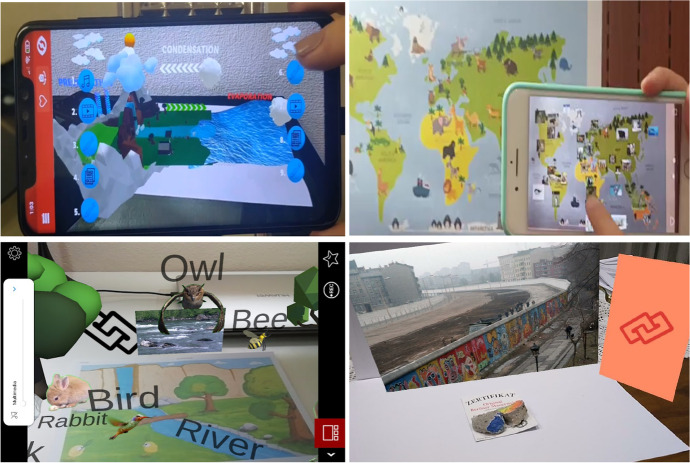
AR projects created by participants for the CLIL classroom: The water cycle (top left), Biodiversity (top right), Wild animals (Bottom left), The Berlin Wall (bottom right)

The research participants created 17 AR projects using different SDKs, the three most popular being Zapworks, Aumentaty and Roar. Each team had to select a specific topic and an educational level (elementary or secondary education) for the CLIL classroom. The AR projects needed to include both discursive (theory) and illustrative (practice) representations through different images or objects (triggers) and multimedia content (overlays). The content should cover various language skills and be properly organized as a sequence of meaningful activities (video lessons, voiceover explanations, online exercises and games) in order to guide the students through the learning process. Therefore, the projects should be clearly based on a lesson plan and carefully designed taking into account the learning goals, both at the content and language level. Appendix A (Fig. 2) shows four images of AR projects created by participants about different topics.

According to the pre-test results, only 4.7% of the teacher candidates had been previous exposed to AR technology in other subjects and none of them had ever used SDKs for AR content creation. Prior experience outside of the classroom stemmed mostly from video games, as 33% of the participants had played Pokémon GO, followed at a distance by Resident Evil, Eyepet, Invizimals, Star Wars Battlefront and Harry Potter Wizards Unite. Concerning AR and VR visualization tools, the most popular were smartphones (74.1%), smart glasses (43.5%), tablets (29.4%) and head-mounted displays (22.4%).

In this experiment, participants received a training session on AR concepts and tools but they required an average of 5–10 hours outside of the classroom to build up their projects. As shown in Table 2, the major hindrances related with SDK usage were: lack of multiuser developing options (#TS8), pricing options (#TS7) and poor technical support (#TS9) although the teacher candidates were moderately satisfied with their projects (#TS10).

**Table 2:** Participants’ level of satisfaction with the SDK and AR project

	N = 85 Cronbach’s alpha: .822	M	SD
#TS1	User-friendly and intuitive interface	3.07	.973
#TS2	Image sensitivity and scan recognition (easily recognizes the images or objects)	3.35	1.043
#TS13	Platform availability and compatibility (iOS, Android, etc.)	3.46	1.150
#TS4	Use of 3D images and tracking (database, import, format, etc.)	3.20	.986
#TS5	Testing & publishing options (projects can be easily uploaded, tested & published)	3.05	1.194
#TS6	(Re)editing options (projects can be easily reedited and modified)	3.09	1.130
#TS7	Cost (free, pricing options, etc.)	2.86	1.264
#TS8	Collaborative development options (supports several users creating the project)	2.18	1.125
#TS9	Technical support availability (video tutorials, on-line support, etc.)	2.92	.991
#TS10	AR visual design and project result	3.65	.996

### Post-test results

Regarding the TPACK results in the post-test, the data analysis provided similar means in the three main sections with small differences, as summarized in Table 3 below and shown in more detail in Appendix A (Table 7). First, the knowledge on CLIL and foreign language teaching principles and practices (CK) yielded the highest score (M = 3.74, SD = .450), although most participants felt more competent in EFL (CK1 M = 4.46, CK2 M = 4.51, CK3 = 4.42) than CLIL (CK4 M = 2.62, CK5 = 2.68). Secondly, the outcome of the Technological Knowledge (TK) section was also positive (M = 3.55, SD = .647), thus demonstrating a moderate self-confidence in their technological skills in general, as evidenced by the scores obtained in some items (TK1 & TK3 M = 3.76, TK4 = 3.72). Lastly, the lowest mean (M = 3.29, SD .648) of the three main sections corresponded to the Pedagogical Knowledge (PK), in other words, participants’ knowledge on different teaching methods and techniques related with CLIL. In this PK section, the teacher candidates took a more moderate position in nearly all items (PK1 M = 3.46, PK3 M = 3.05, PK4 M = 3.35, PK 5 = 3.25).

**Table 3:** TPACK Framework Summary. Results of the main sections and intersections

	N = 85 Cronbach’s alpha .888	N. items	M	SD
TK	Technological Knowledge	6	3.55	.647
CK	Content Knowledge	5	3.74	.450
PK	Pedagogical Knowledge	5	3.29	.648
PCK	Pedagogical Content Knowledge	5	3.04	.782
TCK	Technological Content Knowledge	3	3.34	.727
TPK	Technological Pedagogical Knowledge	6	2.96	.801
TPACK	Technological Pedagogical and Content Knowledge	3	3.05	.776

Compared to the scores obtained in the three main sections the intersections yielded lower results, which is consistent with previous studies on TPACK and EFL among in-service teachers (Tseng, 2016). The mean (M = 3.34, SD = .727) of the TCK (Technological Content Knowledge), i.e. knowledge on how to use AR technology both for visualization and creation, was higher than those of the PCK and TPK, thus revealing the participants’ self-confidence in the new technology. The score was significantly higher as AR users (TCK1 M = 4.02) rather than content creators (TCK3 M = 2.87). This self-perceived competence was confirmed by the results mentioned previously regarding the students’ level of satisfaction with the SDK they employed (Table 2) and the quality of the 17 AR projects they created (Fig. 1). The results of the PCK (Pedagogical Content Knowledge), i.e. knowledge on CLIL methods and practices to teach and evaluate EFL students, were more moderate (M = 3.04, SD .782), particularly regarding the evaluation of the students’ learning progress in the CLIL classroom (PCK 2, M = 2.93).

However, the intersection which provided the lowest outcome (M = 2.96, SD .801) was the Technological Pedagogical Knowledge (TPK), i.e. knowledge on how to use different pedagogical methods and approaches for AR integration in the CLIL classroom. In line with previous research conducted with in-service teachers, this result evidenced that pre-service teachers equally require better training in digitally-oriented pedagogical methods because the lack of this knowledge ‘poses a challenge to many practicing EFL teachers when it comes to dealing with presenting instructional materials via the employment of technology in EFL classrooms’ (Fathi & Yousefifard, 2019, pp. 269–270). Therefore, AR technology needs to be meaningfully integrated in a transformative manner, for example using some of the methods and approaches explained in the introduction (Constructivism, Situated Learning, Connectivism).

The results of the last three items included in the TPACK intersection (M = 3.05, SD .776) evidenced the moderate position adopted by the teacher candidates about their preparation to use different pedagogical methods and integrate AR technology in the CLIL classroom.

The participants’ attitudes toward AR were measured in the post-test through the Augmented Reality Applications Attitude Scale (ARAAS), based on Küçük et al. (2014) and Díaz Noguera et al. (2017). This scale, which includes reverse coding to avoid the acquiescence bias of self-perceived surveys, consisted of 23 items arranged into three dimensions: ‘relevance’, ‘satisfaction’ and ‘reliability’. It was enlarged to include a fourth dimension related to our research on AR integration in the CLIL classroom: ‘beliefs’. The internal consistency of the resulting scale (α = .956) was tested through IBM SPSS 22.0 software as shown in Table 4.

**Table 4:** Attitudes toward AR applications (ARAAS scale)

	N = 85 (α = .956)	M	SD
	**Relevance (α = .875)**		
#1*	I get bored while I use AR applications	2.51	1.042
#2*	It is difficult to use AR applications	2.09	.959
#3	The AR applications of 3D objects provide a feeling of reality	3.86	.804
#4*	Studying the topics is more difficult due to AR applications	2.36	.871
#5	I would like to use AR applications in other topics and modules	3.31	1.047
#6*	The use of AR applications in the classroom is a waste of time	2.06	.864
#7*	AR applications make my learning difficult because they confuse my mind	2.11	.873
#8*	There is no need to use AR applications in the classroom	2.48	1.007
#9	AR improves my opinion about the content of the subject (practical view)	3.53	.765
	**Satisfaction (α = .893)**		
#10	I gain better focus on the topic when AR applications are used	3.38	.845
#11	Demonstration of 3D objects, videos and animations with AR applications increases my curiosity	3.91	.868
#12	I enjoy the classes in which AR applications are used	3.79	.773
#13*	AR applications do not catch my attention	2.24	1.019
#14	Using AR motivates me to work more on a subject	3.31	1.012
#15	I feel more involved in this unit (CLIL and AR) than if I worked in a more theoretical manner	3.39	.965
#16	In general, I think that the use of AR indicates that the teacher is interested in teaching	3.72	.921
	**Reliability (α = .905)**		
#17	I think that this type of AR initiatives would significantly improve the quality of university teaching	3.60	.775
#18	Working with AR allows me to share my ideas, answers and views with my teacher and classmates	3.66	.894
#19	This activity with AR makes me develop other cognitive skills (analysis, synthesis, critical thinking)	3.79	.773
#20	AR has changed my attitude as a student, not only in this class, but generally in language teaching	3.40	.954
#21	The use of AR makes me develop other instrumental skills (handling of tools, information search) in my way of studying	3.78	.864
	**Beliefs (α = .840)**		
#22**	Teachers in a CLIL classroom should be able to use AR in their lessons because it gives a more immersive and authentic experience	3.73	.730
#23**	Teachers in a CLIL classroom should know how to create their own AR-based lessons because it can help students learn better	3.56	.851
#24**	I think the use AR will increase in education over the next years	3.89	.900

*Reverse coding **3 items have been added for research needs (Beliefs)

All four dimensions yielded positive results, which indicates that the teacher candidates clearly support AR adoption for different reasons: the feeling of reality (#3 M = 3.86) and the practical view about the subject content (#9 M = 3.53) in the ‘relevance’ dimension; increased teacher-student interest (#11 M = 3.91 & #16 M = 3.72), enjoyment (#12 M = 3.79) and motivation (#14 M = 3.31), in the ‘satisfaction’ dimension; improved quality of teaching (#17 M = 3.60), collaborative learning (#18 M = 3.66), enhanced cognitive (#19 M = 3.79) and instrumental skills (#21 M = 3,78), in the ‘reliability’ dimension. As pointed out in previous research (Díaz Noguera et al., 2017), the results of AR usage reliability were higher than those of satisfaction and relevance. However, the newly added dimension about ‘beliefs’ clearly outperformed the other three. This indicates that pre-service teachers strongly believe in the need to integrate AR in the CLIL classroom, both for content creation and visualization (#23 & 22), and its increase in education over the next years (#24).

### Data analysis and discussion

In order to evaluate whether any of the subscales of the ARAAS scale, along with other variables measured like participants’ satisfaction with the SDK could account for any between-subject differences in the TPACK scale (i.e. whether attitudes of future teachers toward AR applications according to the ARAAS factors, namely relevance, satisfaction, reliability and beliefs, could predict students’ scores in the main sections and intersections of the TPACK framework), correlational and linear regression analyses were implemented. Also, key assumptions of the linear regression model (normality, linearity, homoscedasticity, and absence of multicollinearity and autocorrelation) were checked and confirmed using the approach recommended by Baños et al. (2019).

First, bivariate correlations were performed to explore relationships between the ARAAS factors, the participants’ previous experience and knowledge on AR technology, their satisfaction with and perceived difficulty of the SDK, and the seven different types of knowledge in the TPACK framework, which were computed as variables. Several statistically significant correlations were found as shown in Table 5. There were consistently positive correlations between the different dimensions of the TPACK framework, as well as between the ARAAS, which highlights the internal consistency of the scales and the intrinsic bonds between the different subdimensions they embody. Besides, positive correlations between Technical Content Knowledge and all of the ARAAS subdimensions were also identified, as well as a negative relation between Technical Knowledge and perceived difficulty of the SDK used (r = −.243, p = .025), which suggests that students having greater ability to use various technologies and associated resources are less likely to find the use of AR tools and applications difficult.

**Table 5:** Influence of AR experience, AR knowledge and ARAAS subdimensions on Technical Content Knowledge

Model		Unstandardized Coefficients		Standardized Coefficients	t	Sig.
		B	Std. Error	Beta		
1	(Constant)	1.319	.447		2.952	.004
	AR experience	−.090	.458	−.019	−.197	.845
	AR knowledge	.844	.404	.212	2.090	.040
	Relevance	.790	.225	.707	3.513	.001
	Satisfaction	−.137	.206	−.135	−.667	.507
	Reliability	−.261	.206	−.262	−1.266	.209
	Beliefs	.165	.131	.164	1.259	.212

*n* = 85; R^2^ = .295; Adjusted R^2^ = .241; *F*(6,78) = 5.447, *p* = .000

Such correlation analyses led up to multivariate linear regressions, which aimed to look into the predictive strength of (i) students’ reported experience in the use of AR tools, (ii) students’ reported knowledge on AR tools, and (iii) any or all of the ARAAS subdimensions (‘relevance’, ‘satisfaction’, ‘reliability’ and ‘beliefs’) on any of the different types of knowledge in the TPACK framework. As shown in Table 5, this model explained 29.5% of the variance (F[6,78] = 5.447, p = .000), although the factors ‘experience in the use of AR tools’ (β = −.019), ‘satisfaction’ (β = −.135), ‘reliability’ (β = −.262) and ‘beliefs’ (β = .164) seemed to have no significant effect on TCK. Specifically, only the factors ‘knowledge on AR tools’ (β = −.019) and ‘relevance’ (β = .707) could account for any differences in the ‘Technical Content Knowledge’ dimension and no other statistically significant effects were found among the measured variables. These results seem to indicate that pre-service teachers’ attitudes toward the use of AR are a predictor of their understanding of how this technology can be used both for visualization and creation. Ultimately, this may indicate that viewing AR technologies as relevant and purposeful helps pre-service teachers build up their skills on how to use them in the CLIL classroom.

A thematic analysis was used for qualitative data obtained through class observation and semi-structured debates. The debates were carried out after the presentations in the last stage and included exploratory questions related to the participants’ level of satisfaction with the AR projects and perceptions about the benefits and limitations of integrating AR in the CLIL classroom. Since all lessons were held online due to Covid-19 restrictions, student presentations and debates were digitally recorded and later transcribed verbatim. Then, the two authors inductively codified the data into several patterns of meaning to identify the main themes. Table 6 contains some verbatim transcriptions. Among the AR affordances, three main aspects emerged: first, enjoyment thanks to the interactive and gamified approach used in the AR projects (S44 and S21); second, relevance as some participants emphasized the sense of reality and immersive experience provided by AR activities which may be helpful to better represent and demonstrate abstract concepts (S81 and S62); third, autonomous learning due to the fact that the AR projects can be shared and completed at any time so that the students have the power to control and regulate their own learning progress (S73).

**Table 6:** Class discussion about AR integration in the CLIL classroom

	**Affordances**
S44	We are very satisfied with our project about the Songhai Empire for the history class. We showed it to our friends and they all felt curious and liked it because it is very interactive with 3D images, activities and on-line games about the history of Africa and they had never done anything like this before.
S21	We enjoyed working on our AR project and preparing the activities. Our lesson about clothes was designed for children around 10 years old and we focused on using different kinds of exercises, so that children don’t get bored and practice different language skills.
S81	I think using AR in the classroom gives you a sense of reality and it can be very helpful to explain some concepts, for example in Mathematics or Natural Sciences.
S62	It took us some time to organize the different activities about the history of Pirates from the easiest to the most demanding, we designed it as a treasure hunt. We started with sea-related vocabulary (island, mast, etc.) and then included some pronunciation and listening activities such as songs and videos on Youtube. We think that children can better understand it through different types of activities and 3D images.
S73	What we liked about the AR project is that we could tell our friends to download the Scope app and scan the image and then they could see the lesson and complete the activities about the British Museum at home. They liked it because we designed it as a virtual tour. We think this can be very helpful for some students learning at home but they may need some technical assistance if they are too small.
	**Limitations**
S24	We tried in the beginning to use ROAR, but we had some problems with image sensitivity and there was a limit of 20 scans for free which was frustrating, so we decided to change to Zapworks. It was more difficult to manage but it had more options.
S37	At first it was a mess because we didn’t know how to create an AR project. Also, it was very hard because none of us had ever used these tools before and we found it difficult because only one person could work at a time so we had to share the email account.
S9	We were lost about how to organize the different activities in our lesson about climate change because we had never learned how to create an AR lesson before and how to use it in a real classroom so we didn’t know how to combine the real teaching with the multimedia activities based on AR with our students.

However, two major limitations were pinpointed by the teacher candidates. Technologically, some participants mentioned certain constraints experienced with the SDK they selected, such as lack of image sensitivity, cloud storage capacity, limited number of scans in the free version, lack of compatibility between different operating systems, limited re-editing options and short-term availability (S24 and S37). These problems are closely related to the fact that AR tools are mostly business-oriented, and although some can be used for educational purposes their free versions have limited functionalities. Pedagogically, the teacher candidates manifested their previous lack of knowledge regarding AR creation and implementation in a real classroom as they had not been trained in technology-oriented pedagogies. Therefore, they ignored how to integrate AR effectively in a CLIL classroom, not for replacing but supplementing other pedagogical materials and to provide the learners with a more lifelike experience. In this sense, the participants advocated for a better training in modern pedagogies where technology is used in a transformative manner (S9). They also expressed some concern about the isolating factor this emerging technology may have among students if used for a prolonged time in the classroom.

Considering the first research question about teacher candidates’ prior AR knowledge, the results evidenced that their experience is mostly based on video games and that their exposure to this emerging technology is limited to the role of AR recipients but not as content creators. This confirms previous studies (Sáez-López et al., 2020; Chang et al., 2020) highlighting the lack of practical knowledge about AR. Initially, the participants were not familiar with the basics of this technology such as key concepts (triggers, overlays) and types (markerless, markerbased, etc.) and its educational applications. However, they demonstrated after the training sessions their skills by using different SDKs to develop their own AR collaborative projects.

Contrary to some studies (Merzlykin et al., 2018) that pinpointed technological constraints as the main obstacle for a meaningful adoption of AR technology in Education, in our research we observed that the major challenge was the lack of expertise among future educators in combining digitally-oriented methods with emerging technologies in the classroom, which corresponds to the Technological Pedagogical Knowledge (TPK) of the TPACK framework. In other words, the teacher candidates easily learned the technological aspects (TK) related with different AR tools but were hesitant about the most appropriate pedagogical methods for their integration (Constructivism, Connectivism, etc) in the classroom. Although mobile AR is widely accessible today, the participants required assistance about how to build their AR lessons taking into consideration certain pedagogical aspects such as content design based on sequential learning and instructional scaffolding. As expressed in previous works (Petrucco & Agostini, 2016), the integration of AR technology in education must be supported by the adoption of an appropriate teaching method, for example Project Based Learning (PBL), which can help the students learn different concepts while they interact with the real and virtual environments.

Regarding the third question, participants’ attitudes toward AR technology were positive at the end of the experiment. This confirms previous results (Chang et al., 2020) which indicated a positive emotional and cognitive engagement of students in the new learning environment, as well as a strong relationship between the motivation of the students and the increase of performance in the academic subject (Cabero-Almenara et al., 2019). Prior studies (Jwaifell, 2019; Jang et al., 2021) highlighted that positive attitudes toward AR and VR-enabled instruction among in-service teachers have an effect on the continuous use of them in the classroom.

Nevertheless, in our study based on the correlation between the TPACK framework and the ARAAS scale we found that early exposure (AR games) and a positive attitude toward AR technology can also be predictors of the digital confidence in AR implementation among future educators. This may contradict previous results (Okumuş, 2021) in which no significant change in pre-service teachers’ self-efficacy level in using AR technology was observed after ten weeks but as the authors of this study stated ‘lack of training on how to use AR technology can decrease users’ self-efficacy in making use of AR technology’ (p.192). Furthermore, the collaborative learning strategy adopted in our experiment helped the teacher candidates share their digital knowledge and improve their skills in designing AR projects.

## Conclusions

Although the implementation of AR technology in Education is in its infancy, this paper has evidenced the need for better pedagogical training among pre-service teachers, particularly in the CLIL classroom which is aimed at learning language and content associatively. In this experiment, the teacher candidates used AR to create their own projects through different SDKs and were post-tested on their self-perceived knowledge of and attitudes toward AR through the TPACK framework and the ARAAS scale. Three main conclusions can be drawn from this research.

First, the TPACK results demonstrated the participants’ higher self-perceived knowledge in the three main sections (CK, TK, PK) compared to the intersections, which can be explained by the fact that the more specific the knowledge becomes the higher the need of adopting a holistic approach. In other words, isolated knowledge about a technology and isolated knowledge about pedagogy are not sufficient to effectively integrate new technologies in the classroom as pointed out by Mishra and Koehler (2006), since teachers often lack the necessary media teaching skills (Bucher et al., 2020). Specifically, the lowest score in the technological pedagogical knowledge (TPK) intersection evidenced the need to provide better training in digitally oriented methodologies (Constructivism, Situated Learning, Connectivism) and practical formation to future educators so that they can learn how to use effectively different techniques to integrate AR in the classroom. In this sense, AR training in education should not only focus on visualization but also on content creation in order to facilitate the adoption of this breakthrough technology for knowledge transformation and not just substitution (Puentedura, 2006), and to empower teacher candidates as AR content designers and not passive recipients.

Second, the results of the ARAAS scale revealed the participants’ positive attitudes in all the scale dimensions: usage reliability, satisfaction and relevance. Consistent with previous works (Bacca et al., 2014), teachers believed that AR can make learning more effective for different reasons: the feeling of reality, increased interest, enhanced cognitive skills, engagement and enjoyment. From the results of the newly added section, the teacher candidates believed in the convenience to integrate AR in the CLIL classroom and its increasing use in education over the next years.

Third, correlational and linear regression analyses demonstrated that students with higher technological skills find the use of SDKs and AR integration easier in the CLIL classroom. This stresses once again the need for better technological and pedagogical preparation among future educators. Besides, pre-service teachers’ positive attitudes toward AR seem to be a predictor of their perception and mastery of AR technology for content creation and visualization. Therefore, positive attitudes, proper training on modern pedagogical methods may be considered as key factors for a meaningful integration of AR in the CLIL classroom. There are three important implications of this study. First and foremost, teacher training programs need to be updated to include practical formation in AR technology, not just for content delivery but also for knowledge transformation through the use of different SDKs and AR types. Then, a holistic approach is necessary for the effective integration of AR technology in Education, covering both technological as well as pedagogical aspects. Last but not least, attitudes toward AR technology among future educators seem to be determined by their previous exposure, which is today mostly restricted to video games, so it is necessary to enhance pre-service teachers’ confidence in AR-integrated instruction in teacher training programs.

The scope of this study may be limited to the research context and participants. A larger sample size may provide more accurate mean values and better detect the effect size of the current study. The results may be also limited to the research instruments, particularly the small number of SDKs used in the AR projects, but participants were free to select those which best fitted their educational projects. More longitudinal studies with longer experimental interventions and students from different educational settings are necessary to identify long-term effects of AR in education. Wide access to digital resources and proper training in transformative technology are key factors to consider when adopting AR technology in the classroom.

The findings are consistent with previous works emphasizing the need to better prepare future educators in emerging technologies. Further research is needed regarding the digital and pedagogical preparation of future educators, and the impact of AR implementation in different CLIL classroom settings in order to measure the impact this breakthrough technology may have on the students’ learning progress.

### Supplementary information

ESM 1 (PDF 667 kb)

## Appendix A

**Table 7:** TPACK results

	TPACK Framework (AR & CLIL) Cronbach’s alpha .951	M	SD
	**Technological Knowledge**		
TK1	I can adjust computer settings such as installing software and establishing an Internet connection	3.76	.984
TK2	I can troubleshoot common computer problems (e.g. printer problems, Internet connection problems, etc.) independently	3.62	.988
TK3	I can use digital classroom equipment such as projectors and smart boards	3.76	.811
TK4	I can create multimedia (e.g. video, web pages, etc.) using text, pictures, sound, video, and animation	3.72	.946
TK5	I can use collaboration tools (wiki, edmodo, 3D virtual environments, etc.) in accordance with my objectives	3.12	.993
TK6	I can learn software that helps me complete a variety of tasks more efficiently	3.29	1.010
	**Content Knowledge**		
CK1	I can express my ideas and feelings by speaking in English	4.46	.568
CK2	I can express my ideas and feelings by writing in English	4.51	.548
CK3	I can understand the speech of a native English speaker easily	4.42	.624
CK4	I can identify different CLIL-related terms and principles (scaffolding, 4 Cs framework, etc)	2.62	.859
CK5	I can mention some of the benefits and challenges of CLIL	2.68	.929
	**Pedagogical Knowledge**		
PK1	I can use teaching methods and techniques that are appropriate for a learning environment in the CLIL classroom	3.46	.716
PK2	I can design a CLIL learning experience that is appropriate for the level of students	2.84	.884
PK3	I can support students’ learning in accordance with their physical, mental, emotional, social, and cultural differences	3.55	.794
PK4	I can reflect the experiences that I gain from my CLIL classes to my teaching process.	3.35	.827
PK5	I can support CLIL students’ out-of-class work to facilitate their self- regulated learning	3.25	.785
	**Pedagogical Content knowledge**		
PCK1	I can manage a CLIL classroom learning environment	3.05	.885
PCK2	I can evaluate CLIL students’ learning progress	2.93	.870
PCK3	I can use appropriate CLIL teaching methods and techniques to support students in developing their language skills	3.00	.873
PCK4	I can prepare CLIL activities that develop students’ language skills	3.09	.868
PCK5	I can adapt a CLIL lesson plan in accordance with students’ language skill levels	3.12	.865
	**Technological Content Knowledge**		
TCK1	I can take advantage of AR and multimedia (e.g. video, slideshow, etc.) to express my ideas about various topics in English	4.02	.707
TCK2	I can benefit from using AR (Augmented Reality) technology to contribute at a distance in the CLIL classroom.	3.13	.936
TCK3	I can use AR authoring tools (SDKs) to create and work collaboratively with other classmates in the CLIL classroom	2.87	1.044
	**Technological Pedagogical Knowledge**		
TPK1	I can meet CLIL students’ individualized needs by using AR lessons	2.94	.850
TPK2	I can lead CLIL students to use AR legally, ethically, safely, and with respect to copyrights	3.11	.951
TPK3	I can support CLIL students as they use AR tools to develop their higher order thinking abilities	3.04	.906
TPK4	I can manage the CLIL classroom learning environment while using AR in the class	2.93	.856
TPK5	I can design CLIL learning materials by using AR-based lessons that support students’ language learning (listening, speaking, reading, etc)	2.88	.837
TPK6	I can design CLIL learning materials by using AR-based lessons that support students’ content learning (Sciences, History, etc)	2.89	.900
	**Technological Pedagogical and Content Knowledge**		
TPACK1	I can use AR-based lessons to support CLILstudents’ language and content learning	2.98	.873
TPACK2	I can support CLIL students as they use AR-based lessons to support their development of language and content skills in an independent manner	3.05	.830
TPACK3	I can support my professional development by using AR authoring tools and resources (wearables) to continuously improve the language and content teaching process.	3.13	.842
