# Supplementary material for: Integrating augmented reality in language learning: pre-service teachers’ digital competence and attitudes through the TPACK framework
Source: Educ Inf Technol (Dordr). 2022 May 27;27(9):12123–46. doi: 10.1007/s10639-022-11123-3 (PMC9136798; doi:10.1007/s10639-022-11123-3)

APPENDIX C . DATA (Not to be included in the article)

Table C1. TPACK results

	TPACK Framework (AR & CLIL) Cronbach's alpha .951	M	SD
	**Technological Knowledge**		
**TK1**	I can adjust computer settings such as installing software and establishing an Internet connection	3.76	.984
**TK2**	I can troubleshoot common computer problems (e.g. printer problems, Internet connection problems, etc.) independently	3.62	.988
**TK3**	I can use digital classroom equipment such as projectors and smart boards	3.76	.811
**TK4**	I can create multimedia (e.g. video, web pages, etc.) using text, pictures, sound, video, and animation	3.72	.946
**TK5**	I can use collaboration tools (wiki, edmodo, 3D virtual environments, etc.) in accordance with my objectives	3.12	.993
**TK6**	I can learn software that helps me complete a variety of tasks more efficiently	3.29	1.010
	**Content Knowledge**		
**CK1**	I can express my ideas and feelings by speaking in English	4.46	.568
**CK2**	I can express my ideas and feelings by writing in English	4.51	.548
**CK3**	I can understand the speech of a native English speaker easily	4.42	.624
**CK4**	I can identify different CLIL-related terms and principles (scaffolding, 4 Cs framework, etc)	2.62	.859
**CK5**	I can mention some of the benefits and challenges of CLIL	2.68	.929
	**Pedagogical Knowledge**		
**PK1**	I can use teaching methods and techniques that are appropriate for a learning environment in the CLIL classroom	3.46	.716
**PK2**	I can design a CLIL learning experience that is appropriate for the level of students	2.84	.884
**PK3**	I can support students' learning in accordance with their physical, mental, emotional, social, and cultural differences	3.55	.794
**PK4**	I can reflect the experiences that I gain from my CLIL classes to my teaching process.	3.35	.827
**PK5**	I can support CLIL students' out-of-class work to facilitate their self- regulated learning	3.25	.785
	**Pedagogical Content knowledge**		
**PCK1**	I can manage a CLIL classroom learning environment	3.05	.885
**PCK2**	I can evaluate CLIL students' learning progress	2.93	.870
**PCK3**	I can use appropriate CLIL teaching methods and techniques to support students in developing their language skills	3.00	.873
**PCK4**	I can prepare CLIL activities that develop students' language skills	3.09	.868
**PCK5**	I can adapt a CLIL lesson plan in accordance with students' language skill levels	3.12	.865
	**Technological Content Knowledge**		
**TCK1**	I can take advantage of AR and multimedia (e.g. video, slideshow, etc.) to express my ideas about various topics in English	4.02	.707
**TCK2**	I can benefit from using AR (Augmented Reality) technology to contribute at a distance in the CLIL classroom.	3.13	.936
**TCK3**	I can use AR authoring tools (SDKs) to create and work collaboratively with other classmates in the CLIL classroom	2.87	1.044
	**Technological Pedagogical Knowledge**		
**TPK1**	I can meet CLIL students' individualized needs by using AR lessons	2.94	.850
**TPK2**	I can lead CLIL students to use AR legally, ethically, safely, and with respect to copyrights	3.11	.951
**TPK3**	I can support CLIL students as they use AR tools to develop their higher order thinking abilities	3.04	.906
**TPK4**	I can manage the CLIL classroom learning environment while using AR in the class	2.93	.856
**TPK5**	I can design CLIL learning materials by using AR-based lessons that support students' language learning (listening, speaking, reading, etc)	2.88	.837
**TPK6**	I can design CLIL learning materials by using AR-based lessons that support students'	2.89	.900

	content learning (Sciences, History, etc)		
	**Technological Pedagogical and Content Knowledge**		
**TPACK1**	I can use AR-based lessons to support CLILstudents’ language and content learning	2.98	.873
**TPACK2**	I can support CLIL students as they use AR-based lessons to support their development of language and content skills in an independent manner	3.05	.830
**TPACK3**	I can support my professional development by using AR authoring tools and resources (wearables) to continuously improve the language and content teaching process.	3.13	.842

Table C2. Correlations between measured variables.

		AR experience	AR knowledge	TK	CK	PK	PCK	TCK	TPK	TPACK	SDK difficulty	SDK satisfaction	Relevance	Satisfaction	Reliability	Beliefs
AR experience	*r*	1	.244*	.082	.050	-.048	-.129	.033	-.106	-.043	.078	.014	.014	-.017	-.017	-.097
	Sig. (bilateral)		.024	.455	.647	.666	.239	.762	.333	.697	.477	.900	.898	.877	.876	.378
AR knowledge	*r*	.244*	1	.250*	.292**	-.008	.010	.223*	.143	.091	-.182	.120	.059	-.012	-.020	-.196
	Sig. (bilateral)	.024		.021	.007	.943	.930	.040	.192	.408	.095	.273	.594	.914	.859	.072
TK	*r*	.082	.250*	1	.413**	.443**	.467**	.481**	.422**	.384**	-.243*	.075	.238*	.219*	.203	.170
	Sig. (bilateral)	.455	.021		.000	.000	.000	.000	.000	.000	.025	.495	.028	.044	.062	.119
CK	*r*	.050	.292**	.413**	1	.420**	.434**	.188	.290**	.216*	-.138	-.026	-.037	-.006	.032	-.010
	Sig. (bilateral)	.647	.007	.000		.000	.000	.085	.007	.047	.208	.811	.737	.957	.773	.928
PK	*r*	-.048	-.008	.443**	.420**	1	.810**	.379**	.659**	.643**	.045	.177	.121	.080	.135	.100
	Sig. (bilateral)	.666	.943	.000	.000		.000	.000	.000	.000	.684	.104	.270	.469	.219	.364
PCK	*r*	-.129	.010	.467**	.434**	.810**	1	.453**	.729**	.694**	-.079	.121	.044	.075	.036	.067
	Sig. (bilateral)	.239	.930	.000	.000	.000		.000	.000	.000	.473	.271	.689	.497	.741	.540
TCK	*r*	.033	.223*	.481**	.188	.379**	.453**	1	.715**	.700**	-.156	.105	.475**	.340**	.319**	.258*
	Sig. (bilateral)	.762	.040	.000	.085	.000	.000		.000	.000	.155	.341	.000	.001	.003	.017
TPK	*r*	-.106	.143	.422**	.290**	.659**	.729**	.715**	1	.905**	-.011	.125	.236*	.245*	.178	.182
	Sig. (bilateral)	.333	.192	.000	.007	.000	.000	.000		.000	.922	.254	.029	.024	.104	.095

TPACK	*r*	-.043	.091	.384**	.216*	.643**	.694**	.700**	.905**	1	.070	.113	.235*	.218*	.165	.173
	Sig. (bilateral)	.697	.408	.000	.047	.000	.000	.000	.000		.522	.303	.031	.045	.132	.112
SDK difficulty	*r*	.078	-.182	-.243*	-.138	.045	-.079	-.156	-.011	.070	1	-.054	-.063	-.055	.024	.053
	Sig. (bilateral)	.477	.095	.025	.208	.684	.473	.155	.922	.522		.623	.569	.620	.827	.630
SDK satisfaction	*r*	.014	.120	.075	-.026	.177	.121	.105	.125	.113	-.054	1	.348**	.270*	.265*	.140
	Sig. (bilateral)	.900	.273	.495	.811	.104	.271	.341	.254	.303	.623		.001	.013	.014	.202
Relevance	*r*	.014	.059	.238*	-.037	.121	.044	.475**	.236*	.235*	-.063	.348**	1	.848**	.836**	.545**
	Sig. (bilateral)	.898	.594	.028	.737	.270	.689	.000	.029	.031	.569	.001		.000	.000	.000
Satisfaction	*r*	-.017	-.012	.219*	-.006	.080	.075	.340**	.245*	.218*	-.055	.270*	.848**	1	.840**	.595**
	Sig. (bilateral)	.877	.914	.044	.957	.469	.497	.001	.024	.045	.620	.013	.000		.000	.000
Reliability	*r*	-.017	-.020	.203	.032	.135	.036	.319**	.178	.165	.024	.265*	.836**	.840**	1	.652**
	Sig. (bilateral)	.876	.859	.062	.773	.219	.741	.003	.104	.132	.827	.014	.000	.000		.000
Beliefs	*r*	-.097	-.196	.170	-.010	.100	.067	.258*	.182	.173	.053	.140	.545**	.595**	.652**	1
	Sig. (bilateral)	.378	.072	.119	.928	.364	.540	.017	.095	.112	.630	.202	.000	.000	.000	

Note: n = 85; *. Correlation is significant at the 0.05 level (2-tailed); **. Correlation is significant at the 0.01 level (2-tailed).

TABLE C3. Linear Regression Assumptions

GET

FILE='C:\Users\Usuario\Desktop\An analysis of pre-

service teachers' digital competence and attitudes through the TPACK framework.sav'. DATASET NAME Conjunto_de_datos1 WINDOW=FRONT.

REGRESSION

/MISSING LISTWISE

/STATISTICS COEFF OUTS R ANOVA

/CRITERIA=PIN(.05) POUT(.10)

/NOORIGIN

/DEPENDENT AvTCK

/METHOD=ENTER AvPrevExp AvPrevKnow AvARRelevance AvARSatisfaction AvARReliability AvARBeli

Regresión

Notas

Resultados creados	
	Conjunto de datos activo Filtro
	Peso
	Tiempo de procesador Tiempo transcurrido Memoria

Resultados creados		
Comentarios		
Entrada	Datos	C:\Users\Usuario\Desktop\An analysis of pre-service teachers' digital competence and attitudes through the TPACK framework.sav
	Conjunto de datos activo	Conjunto_de_datos1
	Filtro	<ninguno>
	Peso	<ninguno>
	Dividir archivo	<ninguno>
	Núm. de filas del archivo de trabajo	8
Tratamiento de los datos perdidos	Definición de perdidos	Los valores perdidos definidos por e usuario se tratarán como perdidos.
	Casos utilizados	Los estadísticos se basan en los casos sin valores perdidos para ninguna variable de las utilizadas.
Sintaxis		REGRESSION /MISSING LISTWISE /STATISTICS COEFF OUTS R ANOVA /CRITERIA=PIN(.05) POUT(.10) /NOORIGIN /DEPENDENT AvTCK /METHOD=ENTER AvPrevExp AvPrevKnow AvARRelevance AvARSatisfaction AvARReliability AvARBelief.
Recursos	Tiempo de procesador	00:00:00,0
	Tiempo transcurrido	00:00:00,0
	Memoria necesaria	9600 bytes
	Memoria adicional requerida para los diagramasde residuos	0 bytes

[Conjunto_de_datos1] C:\Users\Usuario\Desktop\An analysis of pre-service t eachers' digital competence and attitudes through the TPACK framework.sav

Variables introducidas/eliminadas[a]

Modelo	Variables introducidas	Variables eliminadas	Método
1	Average Beliefs mean, Average previous AR experience, Average previous AR knowledge, Average Relevance mean, Average Satisfaction mean, Average Reliability mean[b]	.	Introducir

a. Variable dependiente: Average Technological Content Knowledge

b. Todas las variables solicitadas introducidas.

Resumen del modelo

Modelo	R	R cuadrado	R cuadrado corregida	Error típ. de la estimación
1	,543[a]	,295	,241	,634

a. Variables predictoras: (Constante), Average Beliefs mean, Average previous AR experience, Average previous AR knowledge, Average Relevance mean, Average Satisfaction mean, Average Reliability mean

ANOVA[a]

Modelo		Suma de cuadrados	gl	Media cuadrática	F	Sig.
1	Regresión	13,122	6	2,187	5,447	,000[b]
	Residual	31,317	78	,402		
	Total	44,439	84			

a. Variable dependiente: Average Technological Content Knowledge

b. Variables predictoras: (Constante), Average Beliefs mean, Average previous AR experience, Average previous AR knowledge, Average Relevance mean, Average Satisfaction mean, Average Reliability mean

Coeficientes[a]

Modelo		Coeficientes no estandarizados		Coeficientes tipificados	t
		B	Error típ.	Beta	
1	(Constante)	1,319	,447		2,952
	Average previous AR experience	-,090	,458	-,019	-,197
	Average previous AR knowledge	,844	,404	,212	2,090
	Average Relevance mean	,790	,225	,707	3,513
	Average Satisfaction mean	-,137	,206	-,135	-,667
	Average Reliability mean	-,261	,206	-,262	-1,266
	Average Beliefs mean	,165	,131	,164	1,259

Coeficientes[a]

Modelo		Sig.
1	(Constante)	,004
	Average previous AR experience	,845
	Average previous AR knowledge	,040
	Average Relevance mean	,001
	Average Satisfaction mean	,507
	Average Reliability mean	,209
	Average Beliefs mean	,212

a. Variable dependiente: Average Technological Content Knowledge

```
GRAPH
  /SCATTERPLOT(MATRIX)=AvPrevExp AvPrevKnow AvARRelevance AvARSatisfaction AvARReliability A
  /MISSING=LISTWISE.
```

Gráfico

Notas

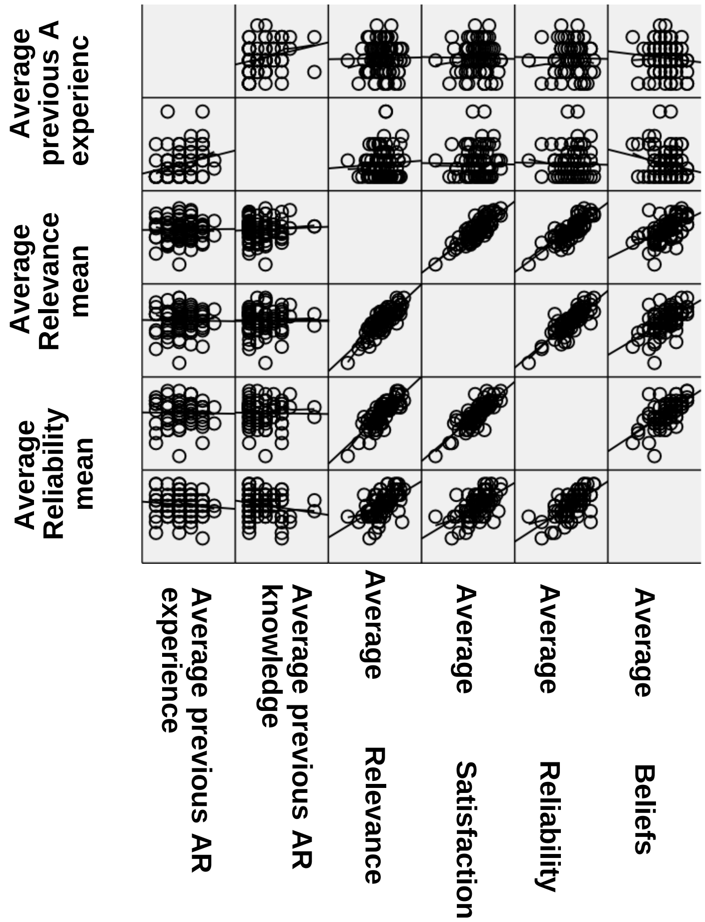

Conjunto_de_datos1] C:\Users\Usuario\Desktop\An analysis of pre-service t eachers’ digital competence and attitudes through the TPACK framework.sav

Resultados creados		01-MAY-2022 00:13:06
Comentarios		
Entrada		C:\Users\Usuario\Desktop\An analysis of pre-service teachers' digital competence and attitudes through the TPACK framework.sav
	Conjunto de datos activo	Conjunto_de_datos1
	Filtro	<ninguno>
	Peso	<ninguno>
	Dividir archivo	<ninguno>
	Núm. de filas del archivo de	8
		5
Si		GRAPH<br>/SCATTERPLOT(MATRIX)<br>=AvPrevExp AvPrevKnow AvARRelevance AvARSatisfaction AvARReliability AvARBelief<br>/MISSING=LISTWISE.
Recursos	Tiempo de procesador	00:00:00,13
	Tiempo transcurrido	00:00:00,11

```
REGRESSION
  /MISSING LISTWISE
  /STATISTICS COEFF OUTS R ANOVA
  /CRITERIA=PIN(.05) POUT(.10)
  /NOORIGIN
  /DEPENDENT AvTCK
  /METHOD=ENTER AvPrevExp AvPrevKnow AvARRelevance AvARSatisfaction AvARReliability AvARBeli
  /SCATTERPLOT=(*ZRESID ,*ZPRED)
  /RESIDUALS DURBIN NORMPROB(ZRESID)
  /SAVE PRED RESID.
```

RegresiónNotas

Resultados creados		01-MAY-2022 00:19:3
Comentarios		
Entrada	Datos	C:\Users\Usuario\Desktop\An analysis of pre-service teachers' digital competence and attitudes through the TPACK framework.sav
	Conjunto de datos activo	Conjunto_de_datos1
	Filtro	<ninguno>
	Peso	<ninguno>
	Dividir archivo	<ninguno>
	Núm. de filas del archivo de trabajo	8
Tratamiento de los datos perdidos	Definición de perdidos	Los valores perdidos definidos por e usuario se tratarán como perdidos.
	Casos utilizados	Los estadísticos se basan en los casos sin valores perdidos para ninguna variable de las utilizadas.
Sintaxis		REGRESSION /MISSING LISTWISE /STATISTICS COEFF OUTS R ANOVA /CRITERIA=PIN(.05) POUT(.10) /NOORIGIN /DEPENDENT AvTCK /METHOD=ENTER AvPrevExp AvPrevKnow AvARRelevance AvARSatisfaction AvARReliability AvARBelief /SCATTERPLOT=(*ZRESID , *ZPRED) /RESIDUALS DURBIN NORMPROB(ZRESID)

		/SAVE PRED RESID.
Recursos	Tiempo de procesador	00:00:00,2[illegible]
	Tiempo transcurrido	00:00:00,2[illegible]

	Memoria necesaria
	Memoria adicional requerida para los diagramas de residuos
[illegible]	PRE

Notas

Notas

	Memoria necesaria	9648 bytes
	Memoria adicional requerida para los diagramas de residuos	256 bytes
Variables creadas o modificadas	PRE_1	Unstandardized Predicted Value
	RES_1	Unstandardized Residual

[Conjunto_de_datos1] C:\Users\Usuario\Desktop\An analysis of pre-service t eachers' digital competence and attitudes through the TPACK framework.sav

Variables introducidas/eliminadas[a]

Modelo	Variables introducidas	Variables eliminadas	Método

1	Average Beliefs mean, Average previous AR experience, Average previous AR knowledge, Average Relevance mean, Average Satisfaction mean, Average Reliability mean[b]	.	Introducir

a. Variable dependiente: Average Technological Content Knowledge

b. Todas las variables solicitadas introducidas.

Resumen del modelo[b]

Modelo	R	R cuadrado	R cuadrado corregida	Error típ. de la estimación	Durbin-Watson
1	,543[a]	,295	,241	,634	2,017

a. Variables predictoras: (Constante), Average Beliefs mean, Average previous AR experience, Average previous AR knowledge, Average Relevance mean, Average Satisfaction mean, Average Reliability mean

b. Variable dependiente: Average Technological Content Knowledge

ANOVA[a]

Modelo		Suma de cuadrados	gl	Media cuadrática	F	Sig.
1	Regresión	13,122	6	2,187	5,447	,000[b]
	Residual	31,317	78	,402		
	Total	44,439	84			

a. Variable dependiente: Average Technological Content Knowledge

b. Variables predictoras: (Constante), Average Beliefs mean, Average previous AR experience, Average previous AR knowledge, Average Relevance mean, Average Satisfaction mean, Average Reliability mean

Coeficientes[a]

Modelo		Coeficientes no estandarizados		Coeficientes tipificados	t
		B	Error típ.	Beta	
1	(Constante)	1,319	,447		2,952
	Average previous AR experience	-,090	,458	-,019	-,197
	Average previous AR knowledge	,844	,404	,212	2,090
	Average Relevance mean	,790	,225	,707	3,513
	Average Satisfaction mean	-,137	,206	-,135	-,667
	Average Reliability mean	-,261	,206	-,262	-1,266
	Average Beliefs mean	,165	,131	,164	1,259

Coeficientes[a]

Modelo		Sig.
1	(Constante)	,004
	Average previous AR experience	,845
	Average previous AR knowledge	,040
	Average Relevance mean	,001
	Average Satisfaction mean	,507
	Average Reliability mean	,209
	Average Beliefs mean	,212

a. Variable dependiente: Average Technological Content Knowledge

Estadísticos sobre los residuos[a]

	Mínimo	Máximo	Media	Desviación típica	N
Valor pronosticado	2,46	4,22	3,34	,395	85
Residual	-1,424	1,717	,000	,611	85
Valor pronosticado tip.	-2,233	2,227	,000	1,000	85
Residuo típ.	-2,247	2,710	,000	,964	85

a. Variable dependiente: Average Technological Content Knowledge

Gráficos

Gráfico P-P normal de regresión Residuo tipificado

Variable dependiente: Average Technological Content Knowledge

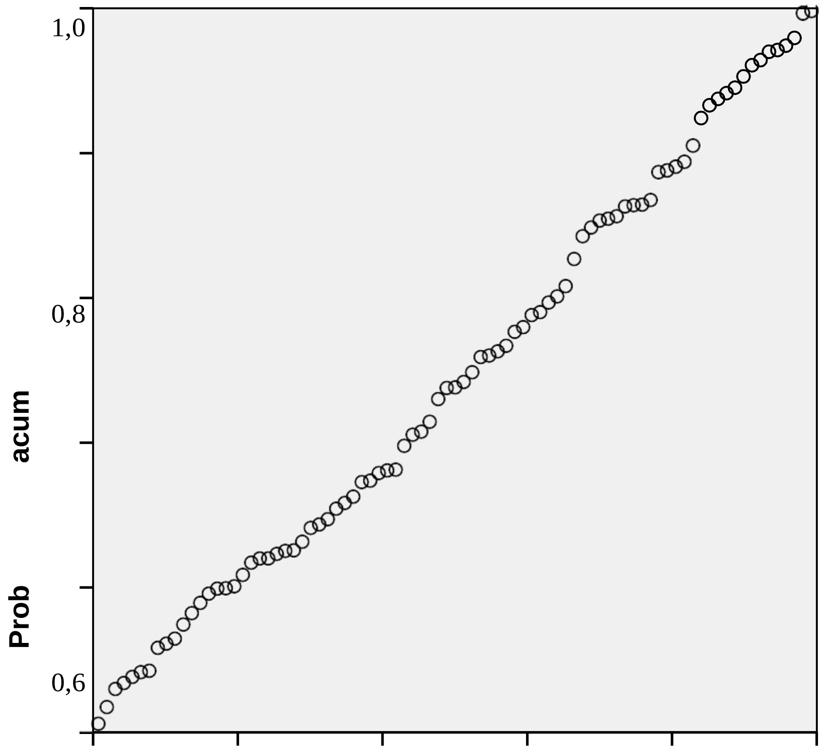

Gráfico de dispersión

Variable dependiente: Average Technological Content Knowledge

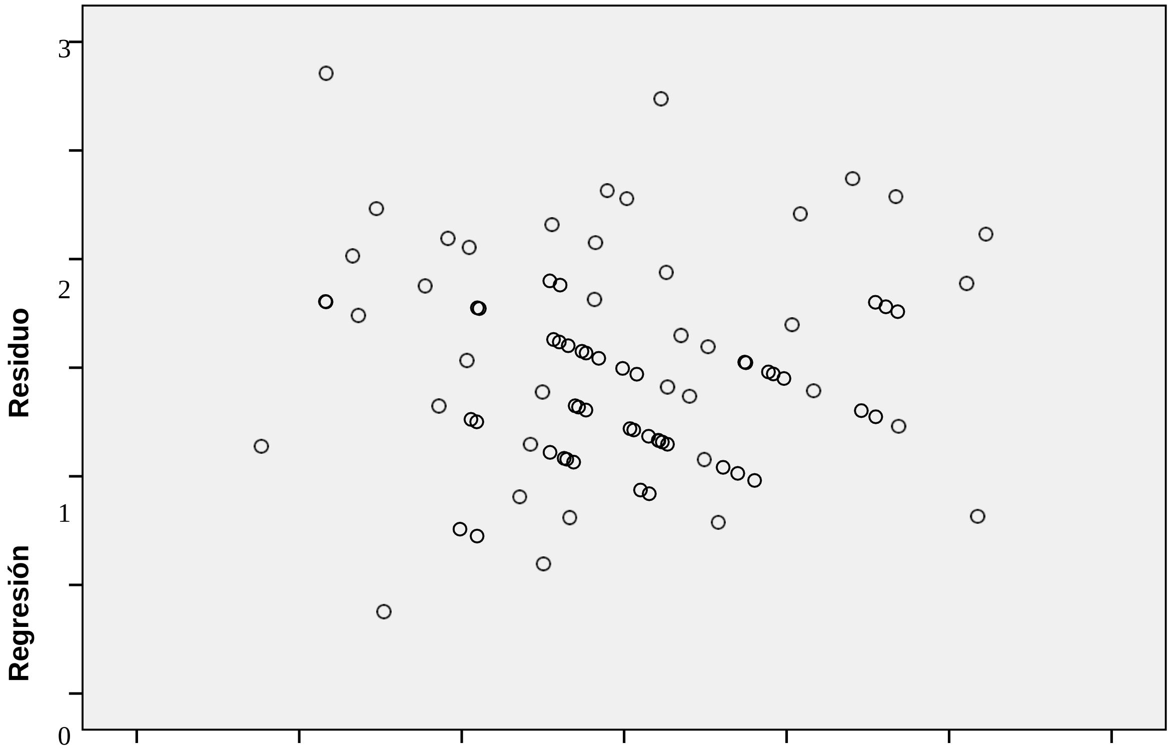

Regresión Valor pronosticado tipificado

```
EXAMINE VARIABLES=RES_1
  /PLOT BOXPLOT STEMLEAF NPPLOT
  /COMPARE GROUPS
  /STATISTICS DESCRIPTIVES
  /CINTERVAL 95
  /MISSING LISTWISE
  /NOTOTAL.
```

Explorar

Resultados creados		
	Conjunto de datos activo	
	Filtro	
	Tiempo de procesador	

Notas

Notas

Resultados creados		01-MAY-2022 00:27:53
Comentarios		
Entrada	Datos	C:\Users\Usuario\Desktop\An analysis of pre-service teachers' digital competence and attitudes through the TPACK framework.sav
	Conjunto de datos activo	Conjunto_de_datos1
	Filtro	<ninguno>
	Peso	<ninguno>
	Dividir archivo	<ninguno>
	Núm. de filas del archivo de trabajo	85
Manipulación de los valores perdidos	Definición de los perdidos	Los valores perdidos definidos por el usuario para las variables dependientes serán tratados como perdidos.
	Casos utilizados	Los estadísticos se basan en los casos que no incluyan valores perdidos en ninguna variable dependiente o factor utilizados.
Sintaxis		EXAMINE VARIABLES=RES_1 /PLOT BOXPLOT STEMLEAF

		NPPLOT<br>/COMPARE GROUPS<br>/STATISTICS DESCRIPTIVES /CINTERVAL 95<br>/MISSING LISTWISE<br>/NOTOTAL.
Recursos	Tiempo de procesador	00:00:00,27
	Tiempo transcurrido	00:00:00,31

[Conjunto_de_datos1] C:\Users\Usuario\Desktop\An analysis of pre-service t eachers' digital competence and attitudes through the TPACK framework.sav

Resumen del procesamiento de los casos

	Casos					
	Válidos		Perdidos		Total	
	N	Porcentaje	N	Porcentaje	N	Porcentaje
Unstandardized Residual	85	100,0%	0	0,0%	85	100,0%

Descriptivos

			Estadístico
Unstandardized Residual	Media		,0000000
	Intervalo de confianza para la media al 95%	Límite inferior	-,1317024
		Límite superior	,1317024
	Media recortada al 5%		-,0124402
	Mediana		-,0375018
	Varianza		,373
	Desv. típ.		,61059564
	Mínimo		-1,42364
	Máximo		1,71693
	[illegible]		[illegible]

Descriptivos

			Error típ.
Unstandardized Residual	Media		,06622840
	Intervalo de confianza para la media al 95%	Límite inferior	
		Límite superior	
	Media recortada al 5%		
	Mediana		
	Varianza		
	Desv. típ.		
	Mínimo		
	Máximo		
	Rango		
	Amplitud intercuartil		
	Asimetría		,261
	Curtosis		,517

Pruebas de normalidad

	Kolmogorov-Smirnov[a]			Shapiro-Wilk		
	Estadístico	gl	Sig.	Estadístico	gl	Sig.
Unstandardized Residual	,066	85	,200*	,989	85	,660

*. Este es un límite inferior de la significación verdadera.

a. Corrección de la significación de Lilliefors

Unstandardized Residual

Unstandardized Residual Stem-and-Leaf Plot Frequency

Stem & Leaf

Frequency		Stem	Leaf
2,00		-1 .	14
15,00		-0 .	555556677788999
28,00		-0 .	0000011112222222333334444444
24,00		0 .	000000111112333333333444
12,00		0 .	556777788999
2,00		1 .	01
1,00		1 .	5
1,00	Extremes		(>=1,7)

Stem width: 1,00000

Each leaf: 1 case(s)

Gráfico Q-Q normal de Unstandardized Residual

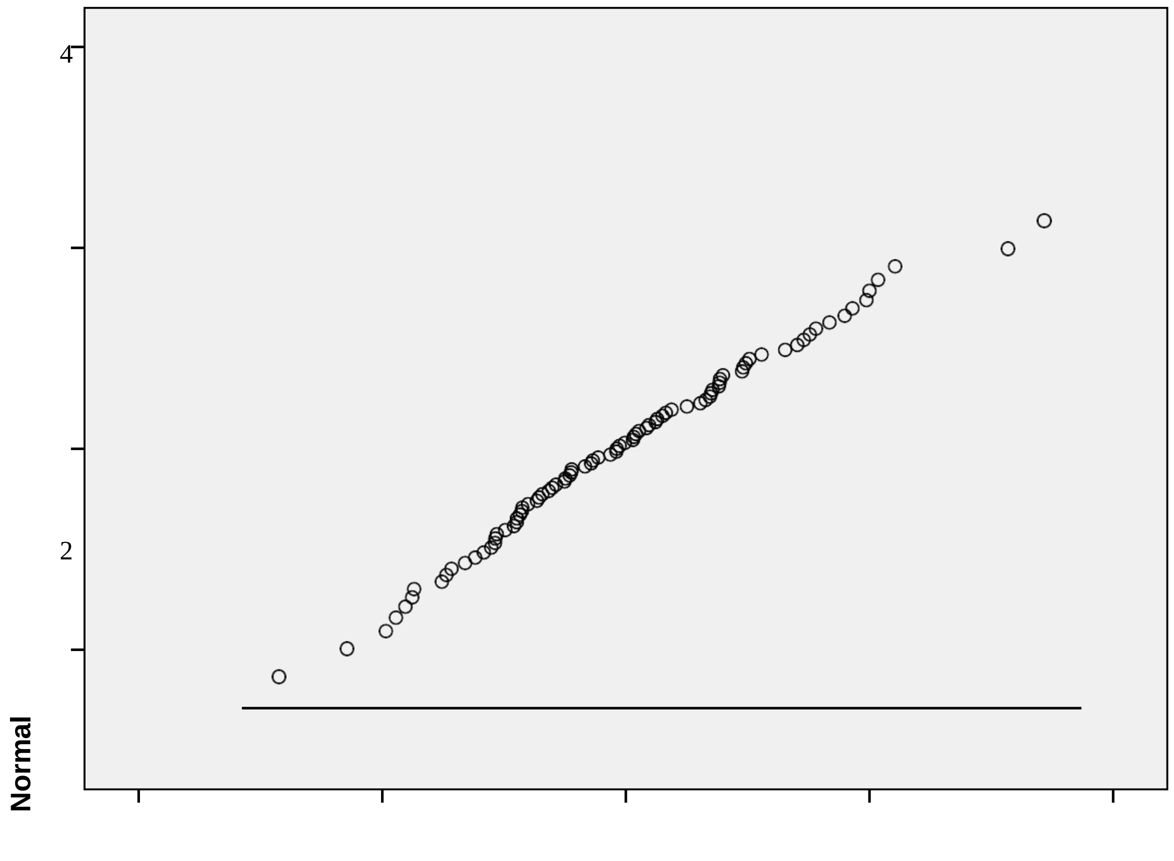

Supplement: Supplementary file 1 — (PDF 667 kb) [file 10639_2022_11123_MOESM1_ESM.pdf]
